# Entamoeba histolytica spliceosome structural components

**DOI:** 10.1099/mic.0.001722

**Published:** 2026-06-22

**Authors:** Israel Canela-Pérez, Odila Saucedo-Cárdenas, Rodolfo Gamaliel Avila-Bonilla, José Manuel Galindo-Rosales, Jesús Valdés

**Affiliations:** 1 Departamento de Bioquímica, CINVESTAV-México, Av. IPN 2508 colonia San Pedro Zacatenco, GAM, CDMX 07360, México; 2 Departamento de Histología, Facultad de Medicina, Universidad Autónoma de Nuevo León, Monterrey, México; 3 Departamento de Genética y Biología Molecular, CINVESTAV-México, Av. IPN 2508 colonia San Pedro Zacatenco, GAMC, DMX 07360, México

**Keywords:** snRNPs, spliceosomal complexes, nuclear-cytoplasmic transport, structural modelling

## Abstract

Discontinuity in eukaryotic genes has been one of the most fascinating discoveries in molecular biology, involving small nuclear RNAs (snRNAs) to remove the intervening non-coding sequences from precursor mRNA transcripts. They assemble with proteins to form a macromolecular complex called a spliceosome. The spliceosome recognizes and removes a single intron while joining flanking exons. The spliceosome’s molecular process can be summarized in four steps: assembly, activation, catalysis and disassembly. During this process, the spliceosome undergoes different reconfigurations that have been characterized biochemically and have recently been structurally addressed mainly in *Saccharomyces cerevisiae*; however, in *Entamoeba histolytica*, the structure of the spliceosome can only be inferred, so in this article, we contribute to the structure of some components that make up this molecular complex in this protozoan parasite.

## Introduction

Most eukaryotic genes are initially transcribed as precursor mRNAs (pre-mRNAs) that contain intervening sequences in the genes (introns), as well as expression sequences (exons). In 1970, it was discovered that introns are removed from pre-mRNAs during splicing, while exons are joined, producing mature mRNAs [1,2]. The processing of pre-mRNAs requires two transesterification reactions catalysed by the spliceosome, which involves ATP hydrolysis through a sequential, regulated process [3].

The spliceosome is a dynamic enzyme that comprises mainly small nuclear ribonucleoproteins (snRNPs), whose biogenesis, assembly and function occur in different cellular compartments. The spliceosome is recruited on the pre-mRNA substrate, and each stage requires entry, repositioning and exit of its components. These changes require the aid of DEAD/DExH RNA helicases and GTPases, which also proofread and perform quality control checks of the first and second transesterification reactions.

**Fig. 1.**
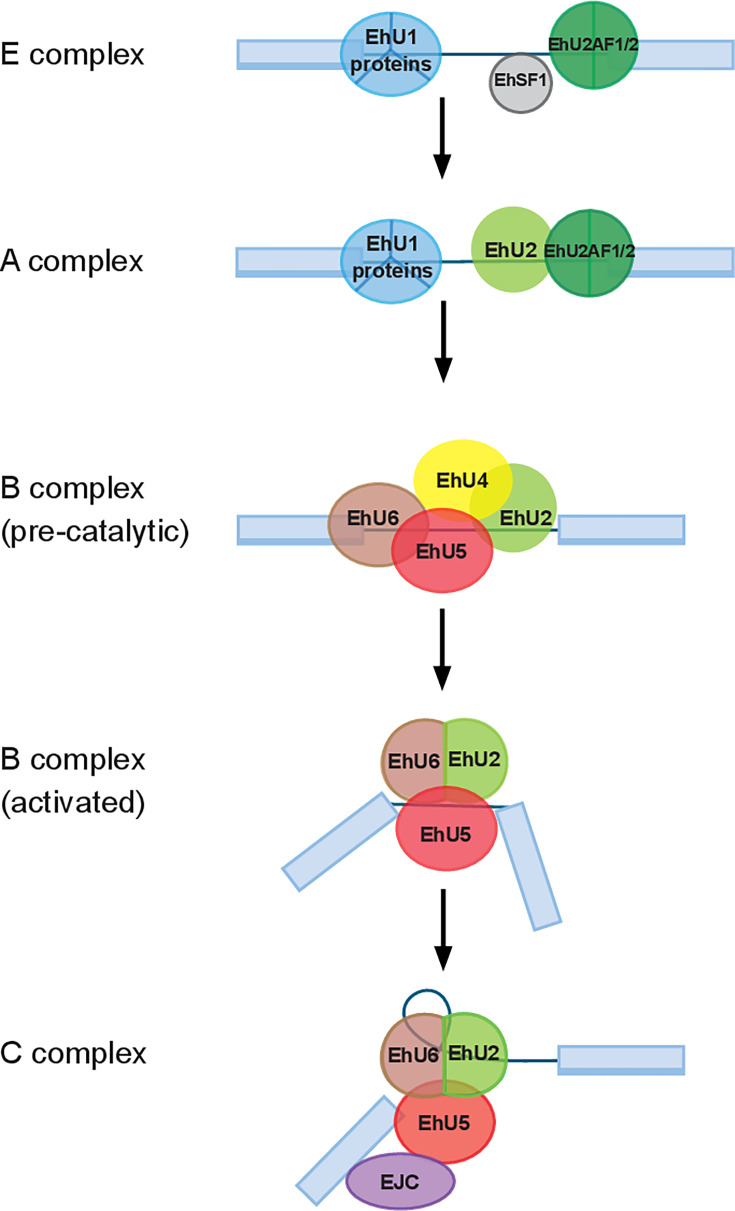
Sequential assembly of protein components during the recruitment of each splicing complex around the pre-mRNA substrate. *E. histolytica* snRNP factors appear colour-coded throughout the figures: U1 snRNP, blue; U2 snRNP, green; U4 snRNP, yellow; U5 snRNP, red; U6 snRNP, salmon. The associated factors of each snRNP are coloured in different shades of their corresponding colour. Colouring of additional complexes: purple for splicing Complex C, shades of lavender for step II factors and exon junction complex (EJC); dark orange for splicing Complex B/B*; and light orange for constitutive Sm and LSm proteins.

First, the U1 snRNP and factors comprising the splicing complex E recognize the different intron elements [the donor 5' splice site (ss), branch point sequence, polypyrimidine (Py) tract and the acceptor 3'ss] required for splicing (Fig. 1). Then, the entry of the U2 snRNP and the exit of splicing factor 1 (SF1) culminate in splicing complex A formation (Fig. 1). In turn, the exit of the U1 snRNP, resulting from the entry of the U4/U6·U5 tri-snRNP and other B complex proteins, yields the splicing complex B (Fig. 1). The entry of a central protein complex (the nineteen complex), the exit of some B complex proteins, the extensive base pairing rearrangements between U2 and U6 snRNAs (forming and placing the catalytic core), the contact of U6 snRNA with the 5'ss and the contact of the U5 snRNA with both exons flanking the intron, the spliceosome becomes catalytically active (B^act^; Fig. 1). Through U6 snRNA mediation, a transesterification reaction occurs between the 2' OH of the A residue in the branch point sequence and the 5'ss, and the entry of factors that will tag the spliced exons (exon junction complex, EJC) for translation, the catalytic complex C is formed (Fig. 1), and the first step of splicing is completed. Next, the second transesterification reaction between the free upstream exon and the 3'ss of the intron lariat-exon intermediary occurs, joining the exons and freeing both the processed mRNA and the intron lariat, while the U4/U6·U5 tri-snRNP remains. Finally, the intron-lariat complex is disassembled, the 3' end tail of the lariat is trimmed up to the branching point, the debranching enzyme hydrolyses the 2'−5' bond, and the linear intron is released.

In this review, we will discuss key components of the spliceosome in *E. histolytica*, previously identified *in vivo*, and, when available, provide structural models that highlight the domains and protein motifs of each spliceosomal protein in this protozoan parasite.

## Transcription of U snRNAS

snRNAs are a family of non-coding RNAs and non-polyadenylated transcripts whose molecular function is carried out in the nucleoplasm. Two main classes of snRNAs are part of the spliceosome: the Sm and Sm-like snRNAs. The U1, U2, U4, U4atac, U5, U11 and U12 belong to the Sm RNA class, while the Sm-like snRNAs are U6 and U6atac. RNA Pol II transcribes Sm snRNAs, with genetic control elements similar to those of the TATA box and enhancer sequences. The initiation of transcription of Sm snRNAs requires binding of the pentameric factor called snRNA-activating protein complex (SNAPc) [4,5]. In addition, Pol II undergoes post-translational modifications in the CTD to enable the binding of these processing factors and their subsequent maturation. The maturation of the snRNA 3' end requires the integrator complex, which binds to the 3' box and cleaves the Pol II transcript [6,7]. The integrator subunits 11 and T9 share sequence similarity with the cleavage and polyadenylation specificity factors subunits 73 kDa and 100 kDa, respectively. Processing the 3' end of Sm-class snRNAs requires a specific promoter, an element located downstream of the cis-acting cleavage site, and several trans-acting factors that bind to the Pol II CTD. The distal and proximal promoter sequence elements of the snRNA genes are equivalent to the enhancer sequences and the TATA box of coding mRNAs, respectively; any substitutions of these elements result in defective 3' end processing of U1 and U2 snRNAs. Pol II recruits the positive transcription elongation factor b to snRNA promoters, which in turn recruits the Little Elongation Complex. In contrast, mRNA promoters recruit the Super Elongation Complex. On the other hand, transcription of snRNAs requires general transcription factors and the SNAPc complex. For snRNAs and mRNAs, 5'-end capping and 3'-end cleavage are thought to occur co-transcriptionally, while processing factors are recruited to nascent transcripts by interacting with the Pol II CTD [8].

In *E. histolytica*, U6 snRNA was the first splicing factor identified and its gene promoter was the first to be characterized [9]. Later, molecular cloning identified the U2, U4 and U5 snRNAs [10]. Notably, the lack of significant homology with eukaryotic U1 snRNAs has led to the conclusion that such snRNAs are absent in amoebas [11] and that the initial spliceosome recruitment relies on 5'ss recognition by the U1 snRNP proteins.

## Assembly of RNPs and Sm/LSm complexes

After the nuclear export of pre-snRNAs to the cytoplasm, dissociation of the CRM1-PHAX-pre-snRNA export complex is carried out by dephosphorylation of PHAX [12]. The survival motor neuron (SMN) recruits the exported snRNAs and combines them with seven Sm proteins to form a toroidal ring containing RNA-binding motifs (RRMs). Sm proteins are delivered to the SMN complex by protein Arg *N*-methyltransferase 5 (PRMT5), which methylates the carboxy-terminal arginine residues of SmB, SmD1 and SmD3 [13,14]. The assembly of Sm proteins protects snRNAs from nucleolytic degradation. However, Sm proteins bind to snRNAs as SmD1-SmD2, SmB-SmD3 heterodimeric subcomplexes or SmE-SmF-SmG heterotrimers. In two crystallography studies, it has been shown that the GEMIN2 complex, a conserved member of the SMN complex in *Homo sapiens* and *Drosophila melanogaster*, binds directly to 5 of the 7 Sm proteins, which then bind to snRNAs [15,16]. In metazoans, the SMN complex has been shown to be involved in the formation of the Sm core RNP; in addition, the SMN-GEMIN2 heterodimer is sufficient for Sm core assembly *in vitro* [15,17, 18].

**Fig. 2.**
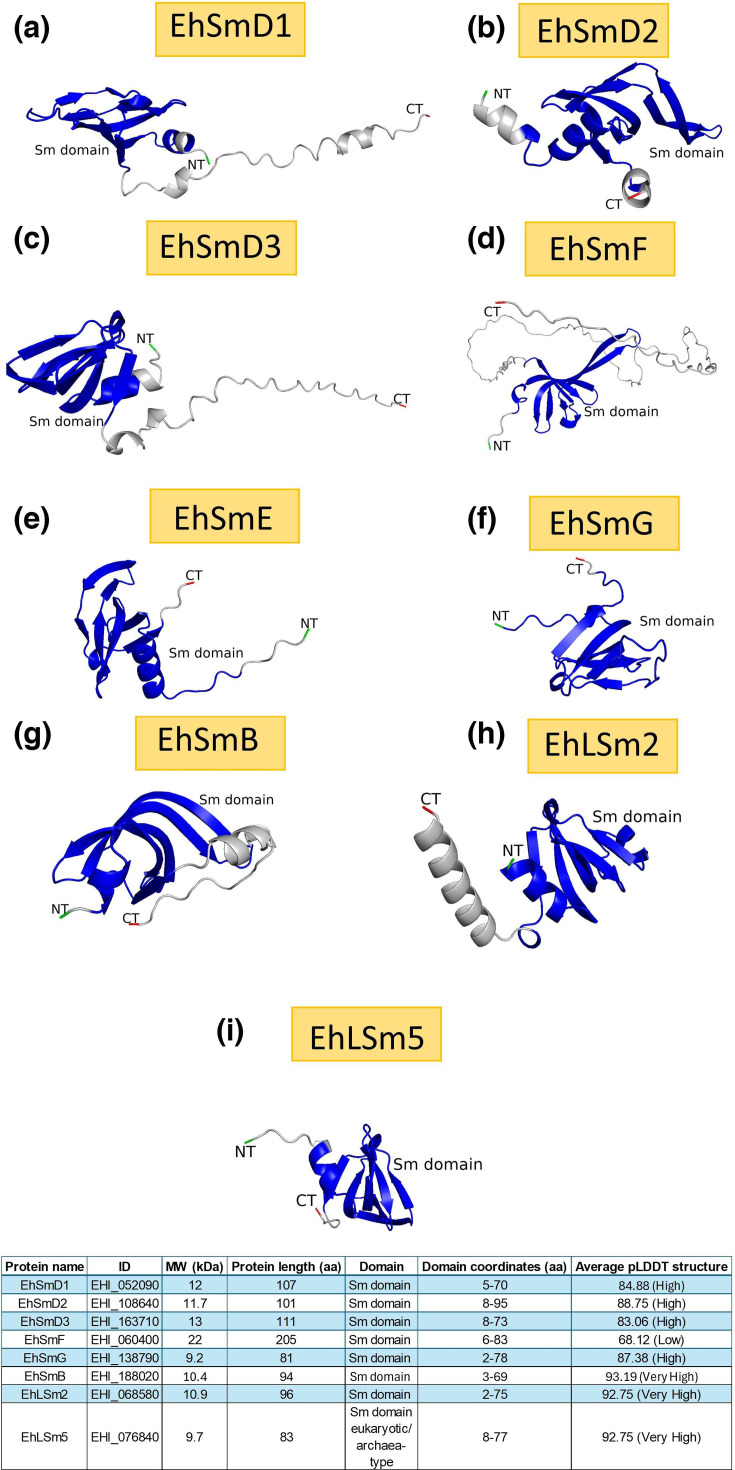
Sm and LSm proteins of *E. histolytica*, where their Sm domain is indicated in blue.

The orthologues of all Sm proteins (EhSmD1, EhSmD2, EhSmD3, EhSmF, EhSmG, EhSmB and EhSmE) have been identified in *E. histolytica* (Fig. 2a–g); due to their well-conserved structural protein domains, it is expected that the assembly of snRNPs takes place similarly to other eukaryotes. Each figure is accompanied by a table listing the relevant domains of the proteins, their gene identifiers, their amino acid positions, their molecular weights and the predicted Local Distance Difference Test (pLDDT) values for their AlphaFold-predicted structures. It must be stressed that although some factors were identified and discussed, their structures could not be predicted due to the lack of corresponding AlphaFold entries. In some cases, models were generated using SWISS-MODEL, and the percentage identity with the template was reported.

The splicing factors discussed here and their orthologues in other Amoebozoa are presented in Table S1 (available in the online Supplementary Material); a comprehensive list of their protein domains appears in Tables S2 and S3 and describes their functions.

## LSm proteins

Members of the LSm protein family are found in all three phylogenetic domains – Eubacteria, Archaea and Eukarya – and are involved in RNA processing and regulation of gene expression. A common feature of LSm proteins is the Sm fold, which consists of five antiparallel β-sheets and one α-helix at the N-terminal. The LSm and Sm proteins participate in spliceosome formation and mRNA decapping. The LSm Hfq protein of bacteria regulates the transcription of different mRNAs through interactions mediated by small regulatory RNAs. Additionally, Hfq is a factor in ribosomal biogenesis, directly structuring the 17S rRNA 3' and 5' sequences by facilitating its processing by RNases. On the other hand, the LSm proteins of Acheobacteria form homoheptamers interacting with U-rich RNA elements [19]. Currently, at least 18 Sm and LSm proteins have been identified in eukaryotic genomes, forming at least six distinct heteroheptameric complexes [20]. These proteins are required for processing snoRNAs, rRNAs, tRNAs, mRNA splicing, histone maturation and mRNA decay [21]. All monomeric LSm proteins are multifunctional. For example, the β4 and β5 sheets of LSm2 can bind various U-rich RNA sequences. Amino acid sequence comparison of the LSm protomers (LSm5, LSm7, LSm4, LSm8, LSm2, LSm3 and LSm6) and the Sm protomers (SmE, SmG, SmD3, SmBB’, SmD1, SmD2 and SmF) shows striking conservation among their respective subunits [22]; the structure of the LSm1-7 complex is nearly identical to that of the LSm2-8 complex [23].

Notably, only two U6 snRNA-associated Sm-Like proteins, EhLSm2 and LSm5, have been identified in *E. histolytica* (Fig. 2h, i). It remains to be seen whether these two proteins can form a functional heptamer around the U6 snRNA, or whether further genome sequencing will reveal the five missing protomers.

## U1 snRNP proteins

Spliceosome assembly is a well-studied molecular event in *S. cerevisiae*, and its steps are conserved in *H. sapiens*. Initially, the U1 snRNP recognizes the 5'ss on the pre-mRNA, initiating complex E formation, and in turn, the U1C protein can join a U1 snRNP subunit [24]. This process is facilitated by the Pol II CTD, which has been reported to interact directly with the U1 snRNP via U1-70k [25,26].

In yeast and humans, a double-helix RNA interaction between U1 snRNA and the 5'ss in complex E is independent of ATP and is stabilized by SR proteins, the cap-binding complex, and U1-70k and U1A binding to the U1 snRNA stem-loops 1 and 2, respectively [27,28].

**Fig. 3.**
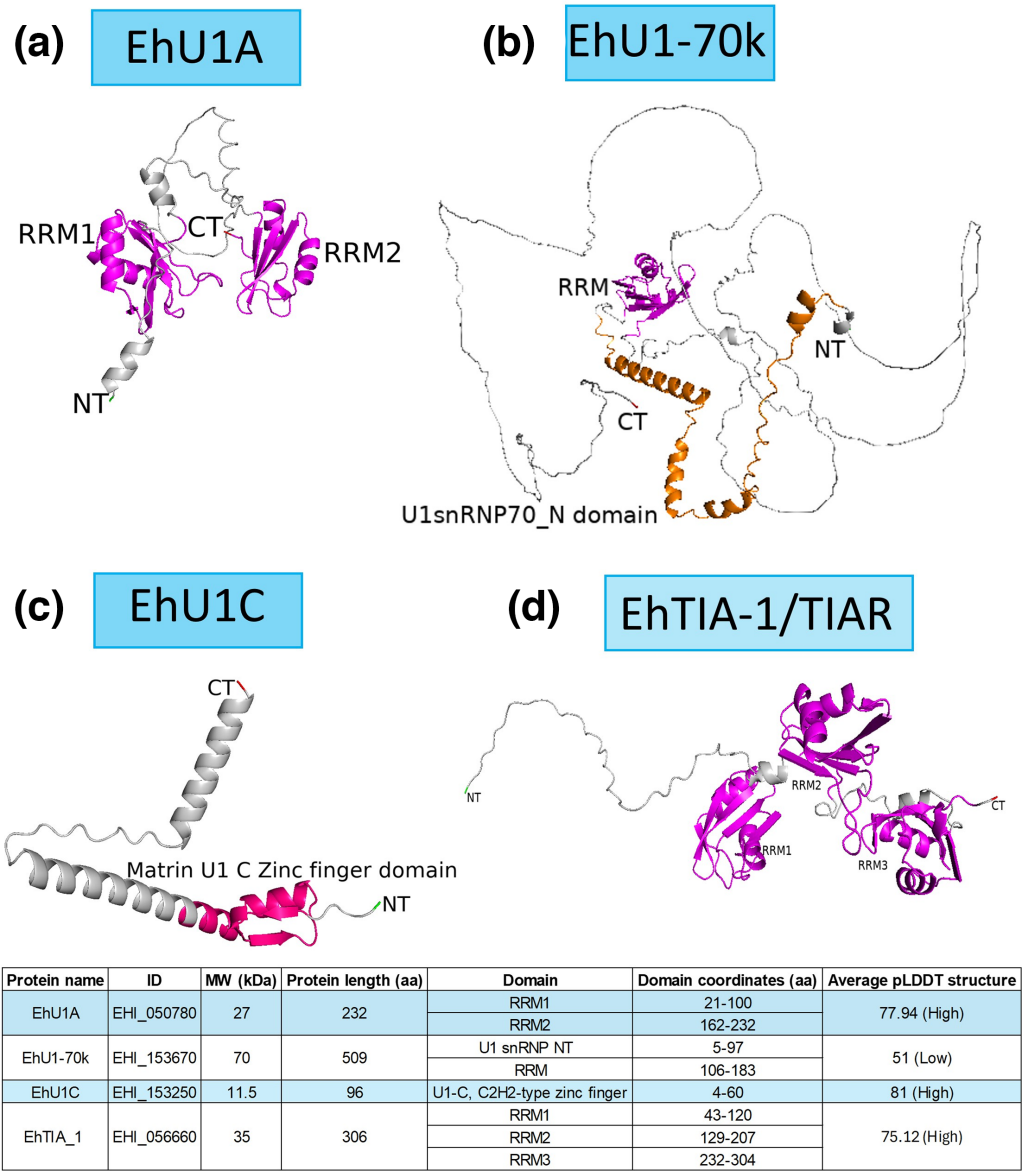
U1 snRNP and U1 snRNP-associated proteins of *E. histolytica,* in which their main domains are shown.

In *E. histolytica,* in the absence of U1 snRNA [11], 5'ss recognition is most likely carried out by EhU1C (our unpublished observations) and EhU1-70k, which probably bind to Pol II, as reported in other eukaryotes [29]. Functional evidence places EhU1A in the early spliceosome [30], even in the absence of a stem-loop II U1 snRNA (Fig. 3a–c).

## U2 snRNP

The U2 snRNP is an essential component of the spliceosome that has a remarkably modular structure, with domains for interaction with U6 snRNA, a site for identification of the intronic branch site (BS) sequence, sites for interaction with large protein assemblies and the Sm-binding site/3' stem-loop [31]. A central protein of the U2 snRNP, Prp8, required for precatalytic, catalytic and post-catalytic stages of the spliceosome, has multiple interactions with the 5'ss, the 3'ss, the catalytic core and various proteins and helicases involved in splicing.

The 3'ss of the introns are recognized by U2 snRNP and its associated factors, such as SF1 and the U2 Auxiliary Factors (U2AFs) that are also components of complex E. Subsequently, in an ATP-dependent process catalysed by DExD/H helicases Prp5 and Sub2, the U2 snRNA recognizes sequences flanking the BS adenosine and interacts with U1 snRNP to form the pre-spliceosome (complex A). Notably, the U2 particle has a high degree of evolutionary conservation; the shorter human U2 snRNA can replace yeast U2 snRNA *in vivo* [32]. In yeast, the U2 snRNA interacts with various proteins that can be grouped into subcomplexes, including SF3a (125 kDa), SF3b (360 kDa) and the snRNA 3'-binding proteins.

**Fig. 4.**
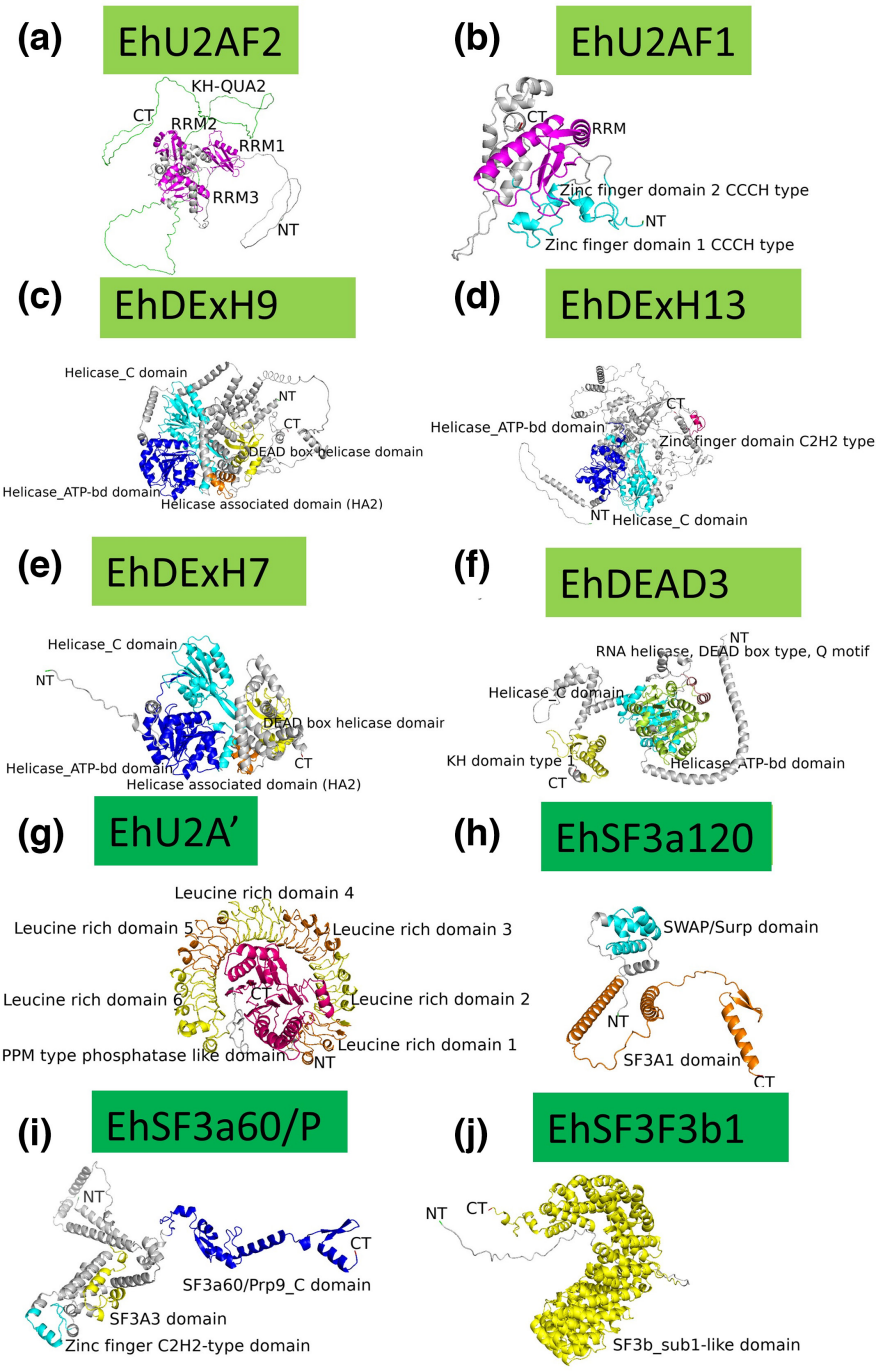

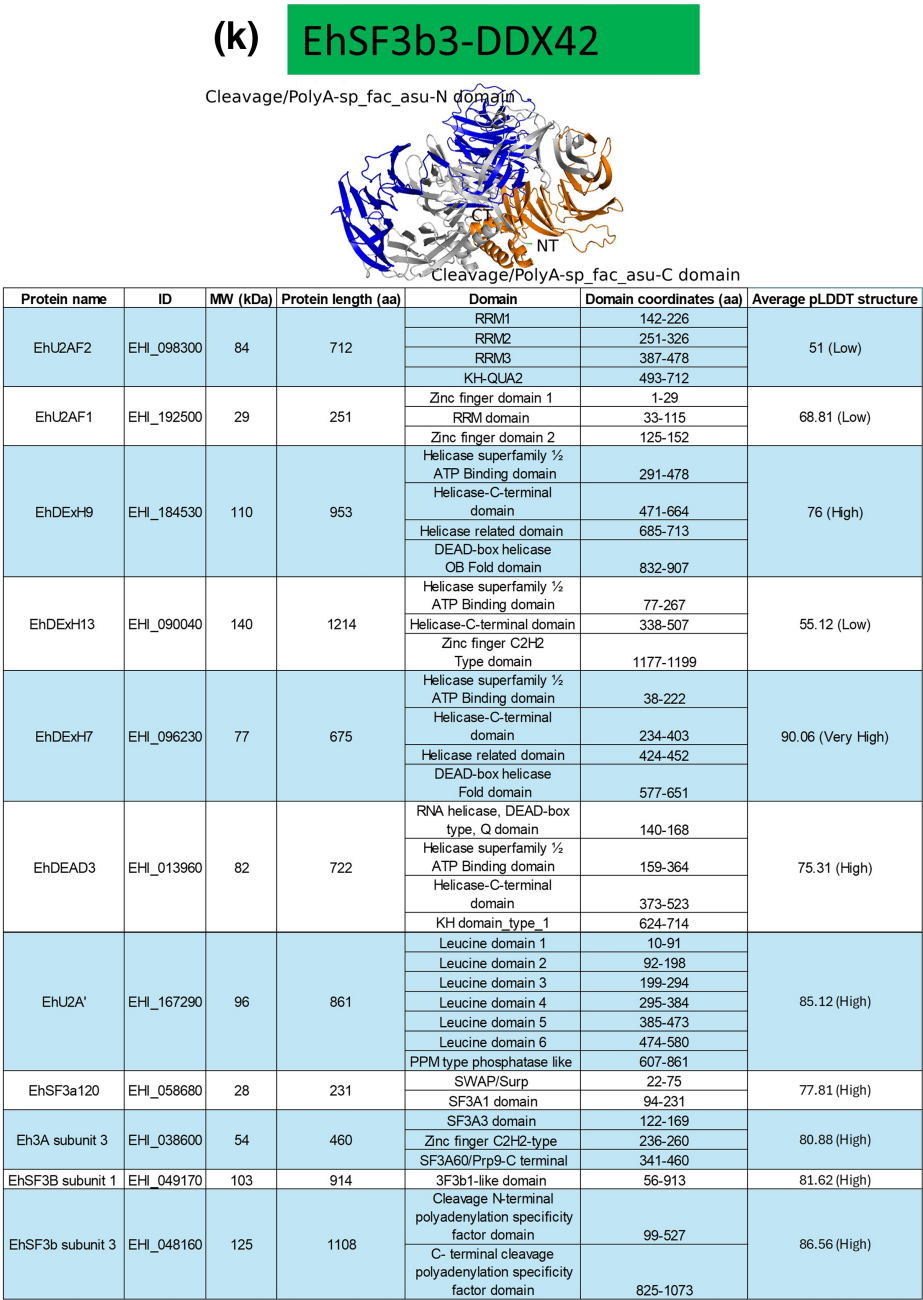
Proteins of the U2 snRNP and U2-related proteins of *E. histolytica* are shown displaying their main domains.

In *E. histolytica,* the U2-related proteins*,* including the U2 snRNP auxiliary factor large and small subunits, EhU2AF2 and EhU2AF1, respectively, are present *in vivo*. They are part of the splicing complex E, which, with the aid of three Prp43 orthologues (EhDExH9, EhDExH13 and EhDExH7) and the Prp5 orthologue EhDEAD3 (Fig. 4a–f), will facilitate transition to complex A.

Also present in *E. histolytica* are EhU2A', EhSF3a120, the splicing factor 3A subunit 3 (EhSF3a60/Prp9), the factor 3B subunit 1 (Eh3F3b1) and factor 3B subunit 3 (EhSF3b3/DDX42) (Fig. 4g, k); all of them are required for complex A assembly.

## U5 snRNP

In yeast and mammals, the U5 snRNP has a loop that interacts with exonic sequences at the 5' and 3'ss [33,34]. The contact between U5 snRNP and the 5' exon is established in the pre-mRNA and persists during the catalytic processes. Such loop anchors to the 5' exon splicing intermediate resulting from the first transesterification reaction and helps align the 5' and 3' exons for the second catalytic reaction [35]. *In vitro*, blocking U5 snRNP assembly to the spliceosome inhibits pre-mRNA splicing, which is restored after the addition of U5 snRNP [36]. The U5 snRNP (PRP8 in *S. cerevisiae*) is conserved in size and sequence across the phylogenetic spectrum, suggesting a key role in the spliceosome [37]. Biochemical analyses of mammalian U5 snRNP and U4/U6-U5 tri-snRNP have shown that they bind a 200 kDa ATP-binding protein that has RNA-dependent ATPase activity [38]. Furthermore, PRP8, its human orthologue and its homologue in *Caenorhabditis elegans* all possess two helicase DEXH domains. The presence of these DEXH motifs suggests that this protein may be involved in one or more conformational changes within the RNA–RNA interaction network of the spliceosome [39,40].

**Fig. 5.**
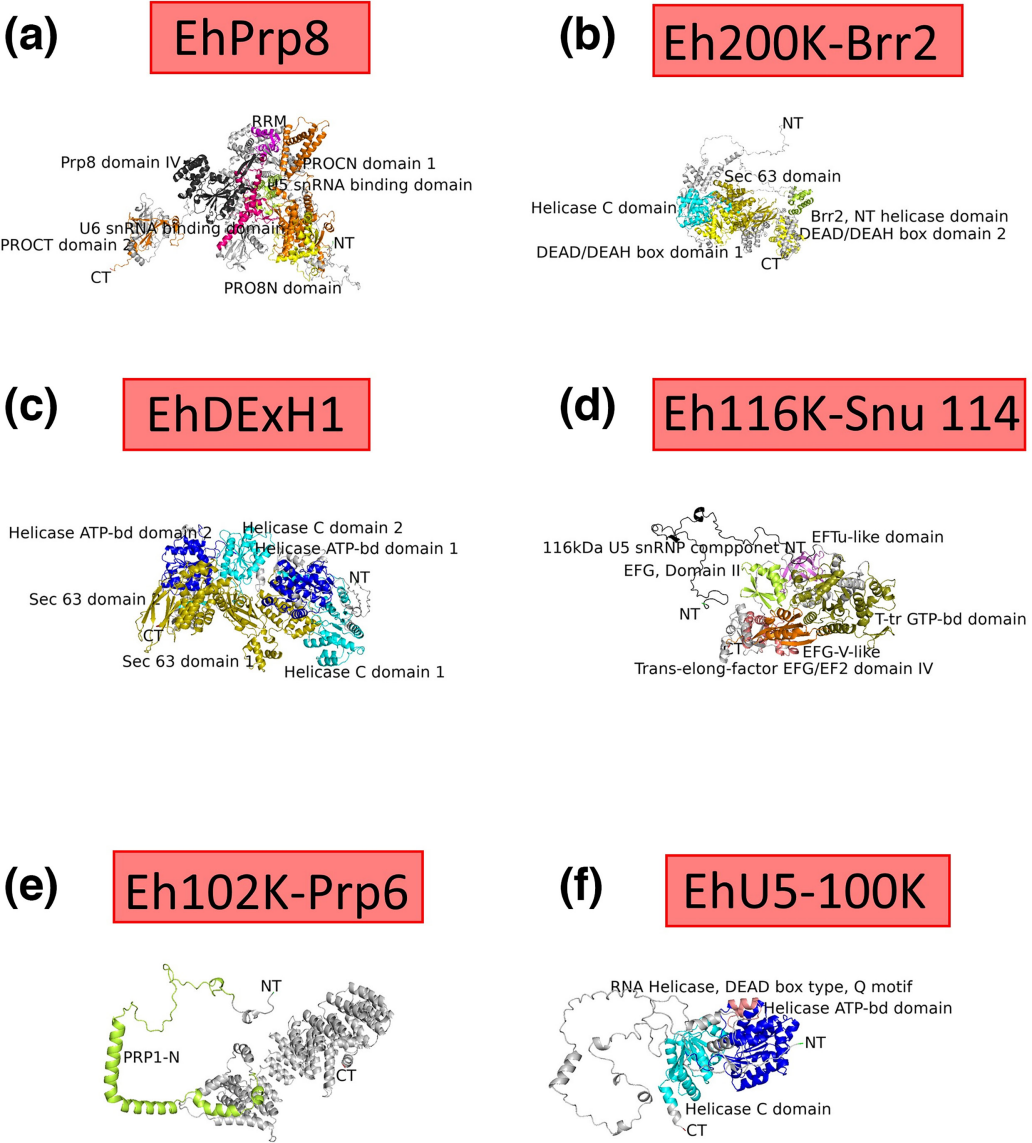

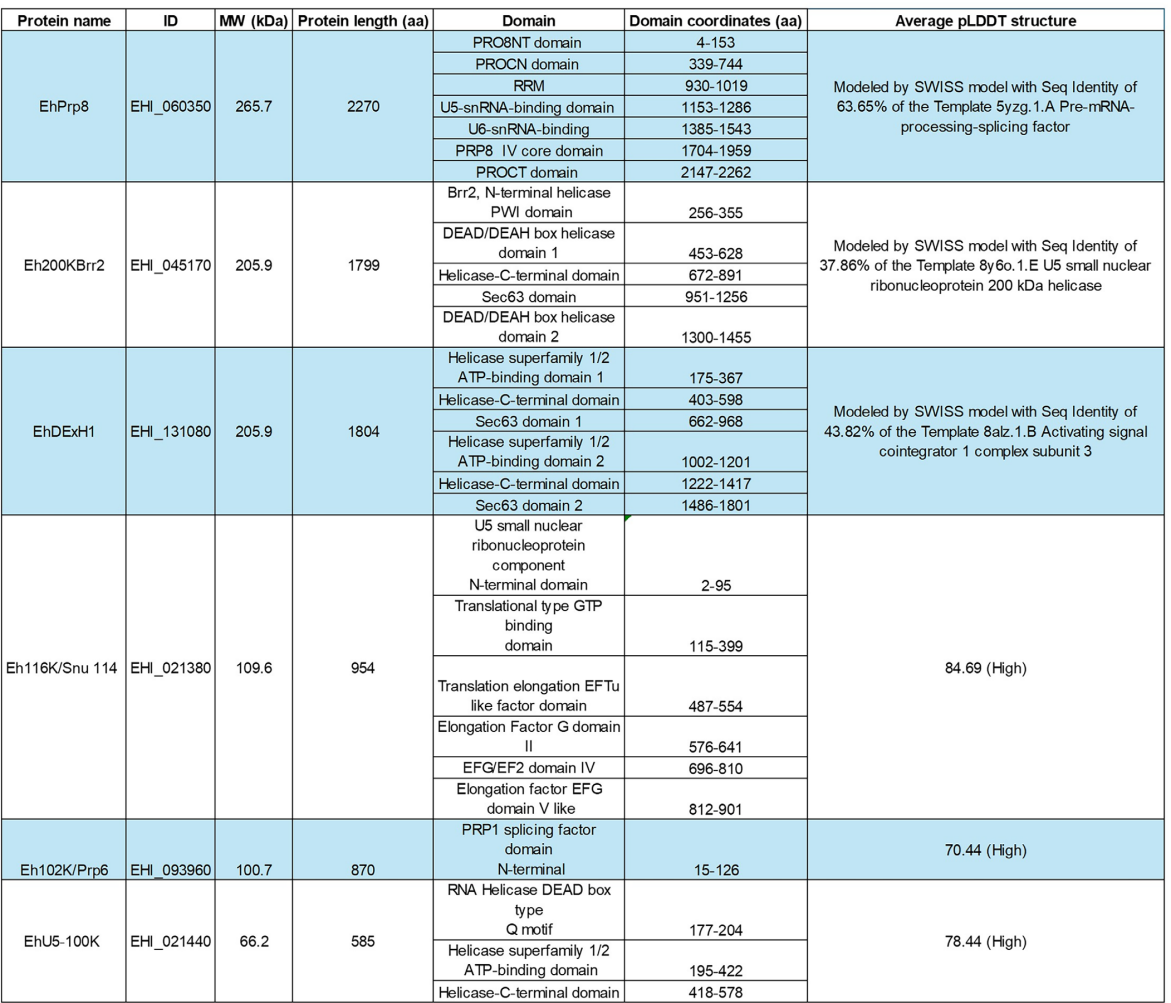
U5 snRNPs proteins of *E. histolytica,* showing their main domains.

In *E. histolytica*, the splicing factor EhPrp8 harbours all the relevant domains required for its central role in splicing and shows a striking conservation with its human orthologue, further confirming its role in splicing. Two orthologues of Brr2 are also present, EhDExH10/U5 and EhDExH1, which are responsible for structuring the fully-assembled precatalytic spliceosome (pre-B Complex) by unwinding the U4/U6 snRNA duplex, and the U5 snRNP subunit protein known as Eh116K/Snu114, which controls the activities of EhDExH10/U5 and EhDExH1. Also present is the tri-snRNP bridging factor Eh102K/Prp6. In the case of EhDEAD4, also known as U5-100K, since no U1 snRNA exists, its function might be restricted to annealing U6 snRNA to the 5'ss, as reported in yeast [41,42] (Fig. 5a–f).

## U4/U6 snRNP

The U4 and U6 snRNAs are primarily found as a single particle, the U4/U6 snRNP. The U4 snRNA contains an Sm-binding site that facilitates its association with snRNPs. On the other hand, U6 snRNA lacks an Sm domain, and its interaction with proteins occurs indirectly via intermolecular interactions with the U4 snRNP [43]. The spliceosomal U4/U6 di-snRNP contains several bases paired between U4 and U6 snRNAs.

Regarding the protein components of the U4/U6 di-snRNP, in *S. cerevisiae*, 18 proteins are present: Snu13, Prp31, Prp3 and Prp4, seven Sm proteins and seven LSm proteins [44]. The pre-formed LSm protein 7 ring binds to the 3' end sequences of the U6 snRNAs, and three Sm protein sub-complexes, SmB-SmD3, SmD1-SmD2 and SmE-SmF.SmG, assemble around the Sm sequence close to the 3' end of the U4 snRNA [45,47]. In yeast, it has been reported that Prp3 and Prp4 are the only specific proteins of the U4/U6 complex, and both proteins form a dimer that binds to the 5' stem loop of the U4/U6 duplex [48,50]. During spliceosome activation, the U4/U6 snRNA duplex dissociates, releasing U6 for subsequent base pairing with the U2 snRNA [51].

**Fig. 6.**
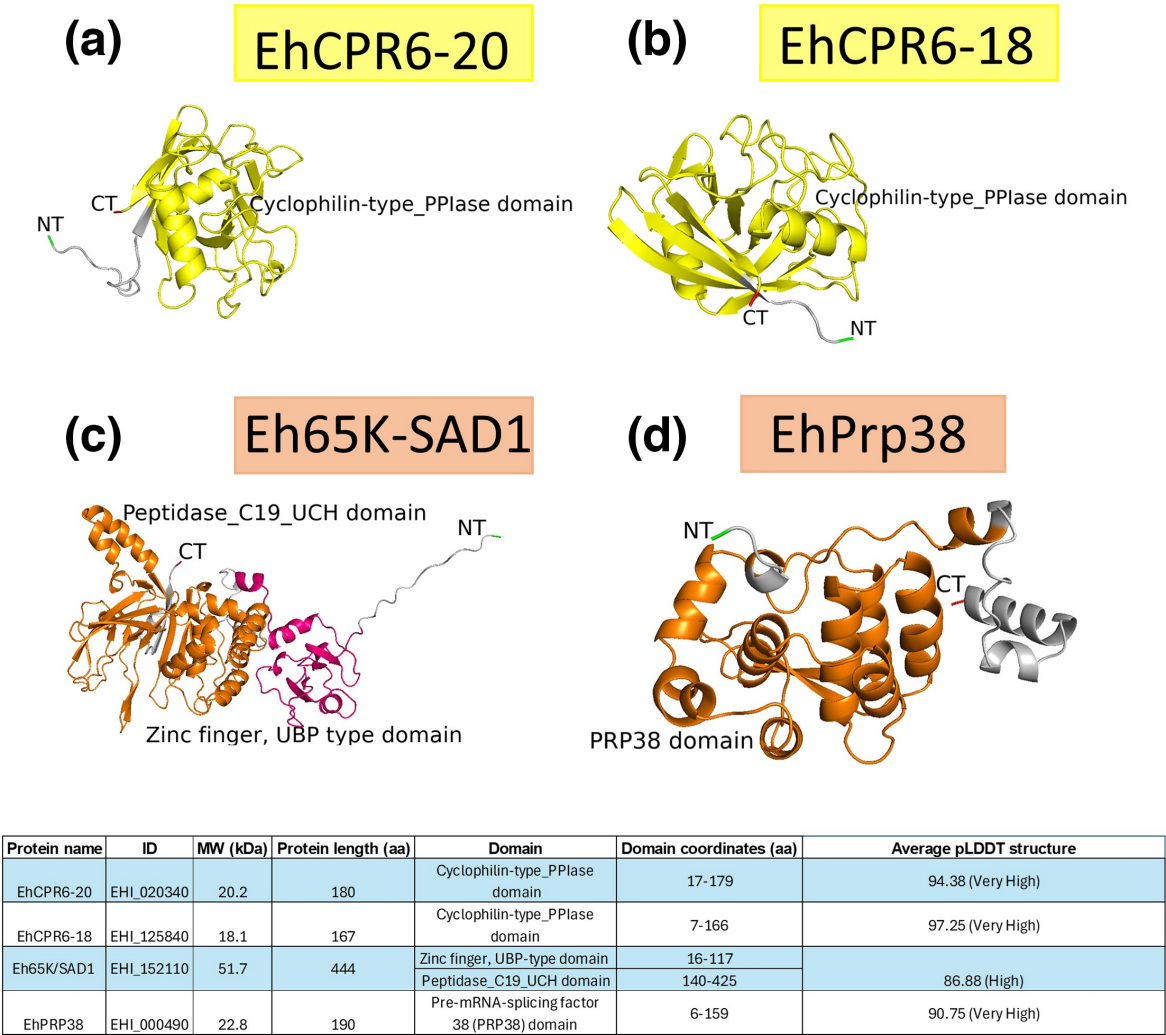
U4/U6 snRNP and U4/U6·U5 tri-snRNP proteins of *E. histolytica* displaying their main domains.

In *E. histolytica*, the EhU6 snRNA gene sequence shows high homology to the consensus sequence of the 50-nucleotide central domain of the RNA molecule. In addition, it contains the conserved ACAGAGA and AGC motifs, which have been shown to be essential for the U6 snRNA catalytic core in the active spliceosome [9,52,54], as are U6 functions in other organisms [55,57]. These motifs are conserved in all species except in *C. fasciculata* [58]. From the protein components of the U4/U6 di-snRNP, we identified only two orthologues of the peptidyl-prolyl-cis-trans isomerase EhCPR6 of 20 and 18 kDa, respectively (Fig. 6a, b).

## U4/U6·U5 tri-snRNP proteins

The U4/U6·U5 tri-snRNP is a 1.5 MDa preassembled complex composed of U5, U4 and U6 snRNAs, as well as more than 30 proteins, including Prp8, Brr2 and Snu114. This is a crucial protein complex required before spliceosome activation. The structure of this complex has been elucidated by cryoEM in *S. cerevisiae* [43,59]. Prp8, Brr2 and Snu114 all play crucial roles in spliceosome activation and active-site formation. Prp8 forms bonds with four thiouridines at specific positions of U5 and U6 snRNAs and residues of the pre-mRNA substrate, indicating that Prp8 is involved in the catalytic RNA core [60,61]. The activity of Brr2 helicase is regulated by GTPase Snu114. Brr2 generates a conformational change of the U4-U6 snRNA duplex [62]. The U4/U6·U5 tri-snRNP can be recruited to the exon-defining complex, which can be directly transformed into an intron-crossed type B complex without prior formation of an intron-crossed A complex [63]. Furthermore, the interaction of the U4/U6·U5 tri-snRNP with the Cap-binding complex facilitates splicing [64], confirming the notion of co-transcriptional splicing.

In *E. histolytica*, in addition to EhPrp8, EhBrr2 and (of which no full-length human structures are disclosed), components of the tri-snRNP are Eh65K/SAD1, EhPRP38 (Fig. 6c, d) and EhSnu114 (Fig. 5d). While Eh65K/SAD1 is essential for the assembly of the pre-catalytic complex, EhPRP38 regulates the assembly and destabilization of factors during spliceosomal activation, and the GTPase EhSnu114 helps drive the conformational changes exerted by EhBrr2 for efficient U4 snRNA release, leading to the catalytic core conformation by hybridization of the U2 and U6 snRNAs, while the BS is still tethered to the mid part of the U2 snRNA.

## Splicing complex E to complex A transition

The E complex is established when the 5'ss and the 3'ss are defined by respective splicing factors. The 5'ss of amoebic introns would be recognized by the U1 snRNP proteins U1C and U1-70k, and the most peripheral U1A. In some cases, the recognition of either 5'ss is aided by the accessory protein EhTIA-1/TIAR (Fig. 3d). The closely related RNA-binding proteins TIA-1 and TIAR (TIA-1/TIAR) have been implicated in apoptosis and other nuclear functions. TIA-1/TIAR shares 46% identity with their orthologues in *D. melanogaster* and *Caenorhabditis elegans*, indicating evolutionary and functional conservation [65]. In the nucleus, TIA-1 activates the splicing of specific pre-mRNAs and some 5'ss of exogenous genes. Also, TIA-1/TIAR activates the splicing of alternative exons with weak 5'ss followed by U-rich stretches of endogenous pre-mRNAs. Furthermore, TIA-1 directly activates the 5'ss of TIAR’s alternative, suggesting that both proteins can self-regulate their expression at the splicing level. Interestingly, when the U-rich sequence following one of the 5'ss activated by TIA-1 or TIAR is removed, the 5'ss in question is no longer used, and another 5'ss is used. These other 5'ss are also followed by a U-rich sequence, strongly suggesting that a U-rich region immediately downstream is necessary for TIA-1 activation of a 5'ss [66]. *In vitro*, *D. melanogaster* msl-2 pre-mRNA splicing requires TIA-1 in the presence of a 5'ss followed by a U-rich sequence [67]. In *E. histolytica*, EhTIA-1/TIA was detected *in vivo* [30], suggesting its involvement in the splicing of Entamoeba pre-mRNAs.

In turn, the 3'ss would be defined by EhSF1, EhU2SF2 and EhU2SF1 binding to the BS, the Py tract and the AG dinucleotide of the 3'ss, respectively. These proteins interact with each other via UHM and ULM motifs, enhancing the binding affinity to the mRNA [68]. Whereas the UHM of U2AF1 interacts with the ULM in the N terminus of U2AF2, forming a stable U2AF1/2 heterodimer, the UHM (RRM3) of U2AF2 interacts with the ULM at the end of phosphorylated SF1.

Transition to complex A involves ATP-dependent rearrangements in which the SF1-BS interaction is replaced by the RNA hybrid formed between the U2 snRNA and the BS, and the SF1-U2AF2 interaction is replaced by the SF3B1-U2AF2 interaction [69]. In *E. histolytica*, all of these factors have been identified, and all have conserved UHM and ULM motifs [70]; therefore, a similar scenario is expected in this parasite. Most likely, in *E. histolytica*, the RRM1 and RRM2 domains of EhU2AF2 recognize the Py tract and the EhU2AF2 UHM motif interacts with the ULM motif at the N-terminus of EhSF1 as previously reported [69]. Furthermore, EhU2AF2 contains an unusual KH-QUA2 (84KQ) motif that interacts with EhSF1, thereby increasing their proximity. While the EhU2AF2-UHM stabilizes the binding of EhSF1 to the BS, the 84KQ appears to grip this interaction, blocking the E-to-A transition of the complex, thus inhibiting splicing and negatively affecting the virulence of the parasite [70]. In agreement with this, EhU2AF2 and a paralogue of EhSF3b1 are upregulated in amoebic liver abscesses. Furthermore, the overexpression of the *E. histolytica* orthologue of Syf1/2, EhSyf, one of the central components of the U2 snRNP’s nineteen complex (Prp19/CDC5L complex, discussed below), directly impacts splicing in a general manner [71], reflecting the direct interaction between the U2 snRNP and EhU2AF2, which binds to the Py tracts upstream of the 3'ss and tethers the pre-mRNA/spliceosome to the CTD.

In addition, while EhDEAD3 (Prp5) might be involved in U2 snRNA/BS hybrid formation, the Prp43 orthologues EhDExH9, EhDExH13 and EhDExH7 might be involved in the release of U2, U6 and U5 from the intron lariat post-splicing complex (Fig. 4c–f).

## B/B^act^ complex

Sequential conformational changes occur during maturation of the precursor spliceosome (pre-B complex), to the precatalytic spliceosome (B complex) and the activated spliceosome (B^act^ complex). These transitions are driven by the ATPase/helicases Prp28 and Brr2, respectively. The B complex lacks a functional active site and cannot proceed to the branching reaction. The remodelling of the B complex by RNA-dependent ATPase/helicase Brr2 results in the dissociation of U1 and U4 snRNPs and the recruitment of ~20 proteins to form the B^act^ complex [72,73]. Currently, cryoEM structures of the pre-B and B complexes have been reported in humans. In the pre-B complex, snRNPs U1 and U2 associate with two edges of the tri-snRNP U4/U6·U5. In the case of complex B, the U1 snRNP and Prp28 are dissociated, and the 5' exon is anchored to loop I of the U5 snRNA. On the other hand, the 5'ss is recognized by the U6 snRNA within the duplex formation. In yeast, most of the U2 snRNP and tri-snRNP components, exerted by Brr2, undergo pronounced rearrangements in the transition from pre-B to B complex when compared with the human complexes [74,75]. Complex A associates with the tri-snRNP U4/U6·U5 to generate the pre-B complex, and subsequently, the pre-B complex undergoes RNP remodelling to become complex B, which becomes the B^act^ complex [76]. Additionally, the CryoEM-derived structure of the human B^act^ complex revealed 52 protein components, the U2, U5 and U6 snRNAs and a pre-mRNA [77].

Essential for B-complex activation is the multimeric Prp19/CDC5L core, in which Prp19 serves as the central platform protein [78,79]. Prp19 is found in yeast and human cells as a stable heteromeric complex with CDC5L [80]. Mass spectrometry studies of the Prp19/CDC5L complex indicate that it is made up of seven proteins: Prp19, CDC5L, PRL1, AD002, SPF27, CTNNBL1 (β-catenin 1-like) and HSP73 [81]. Comparative proteomics analysis of spliceosomal complexes A, B and C in *H. sapiens* shows that Prp19/CDC5L interacts with the spliceosome before its catalytic activation; however, its association is stabilized during the transition from complex B to C [82,84]. Immuno-depletion and complementation assays of the Prp19/CDC5L complex in nuclear extracts from HeLa cells show that this complex is required for pre-mRNA splicing before the first catalytic step [81]. Consistent with these data, the Prp19/CDC5L complex stably interacts with the U5 snRNP within the spliceosome and remains associated throughout the catalytic process. Finally, Prp19/CDC5L complex is released from the spliceosome as part of the 35S U5 snRNP, which is thought to be an intermediate disassembled from the post-spliceosomal complex U2, U5 and U6 containing complex [79,82]. The 35S complex has been resolved by CryoEM, showing the presence of the Prp19 complex and its related factors [85].

**Fig. 7.**
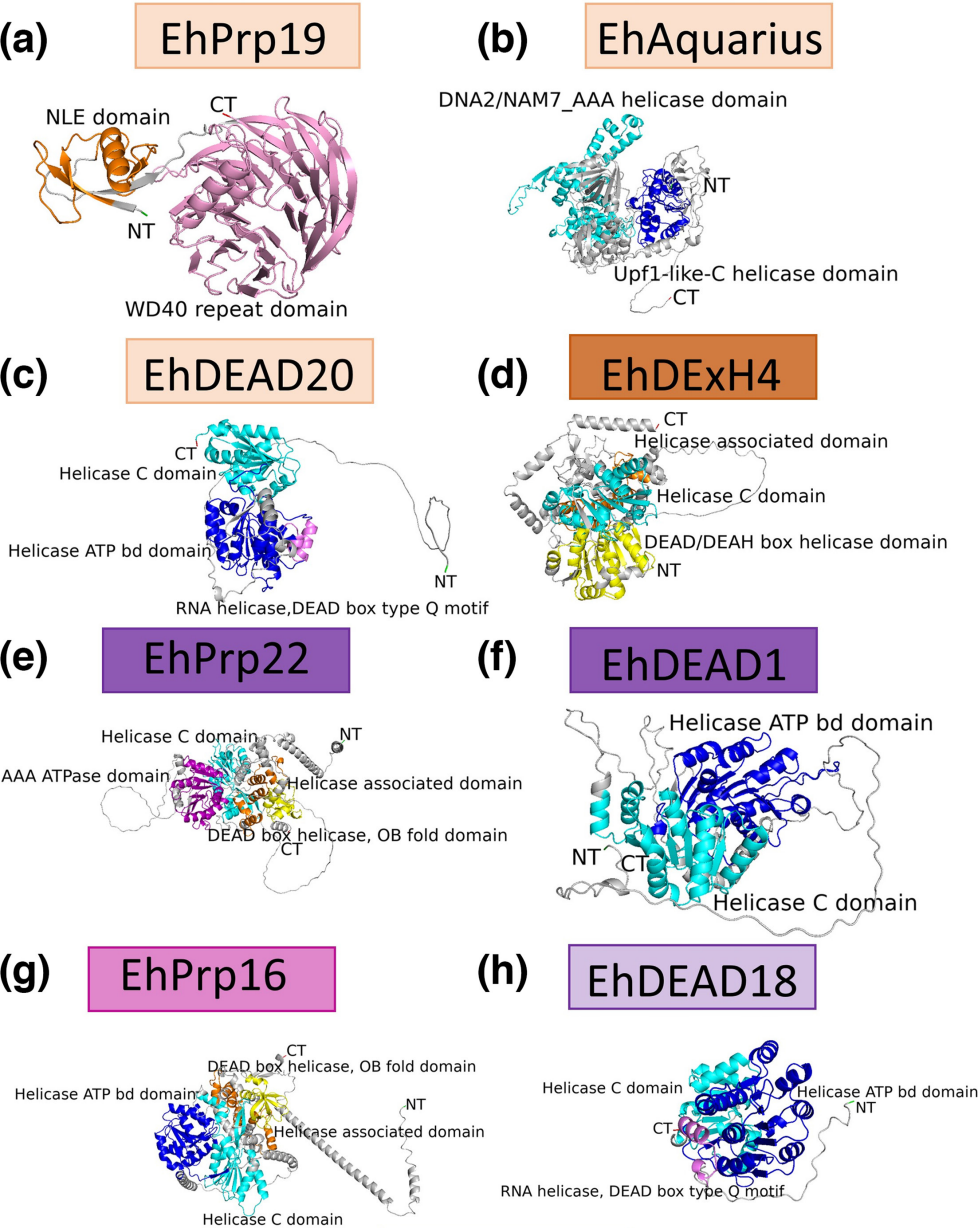

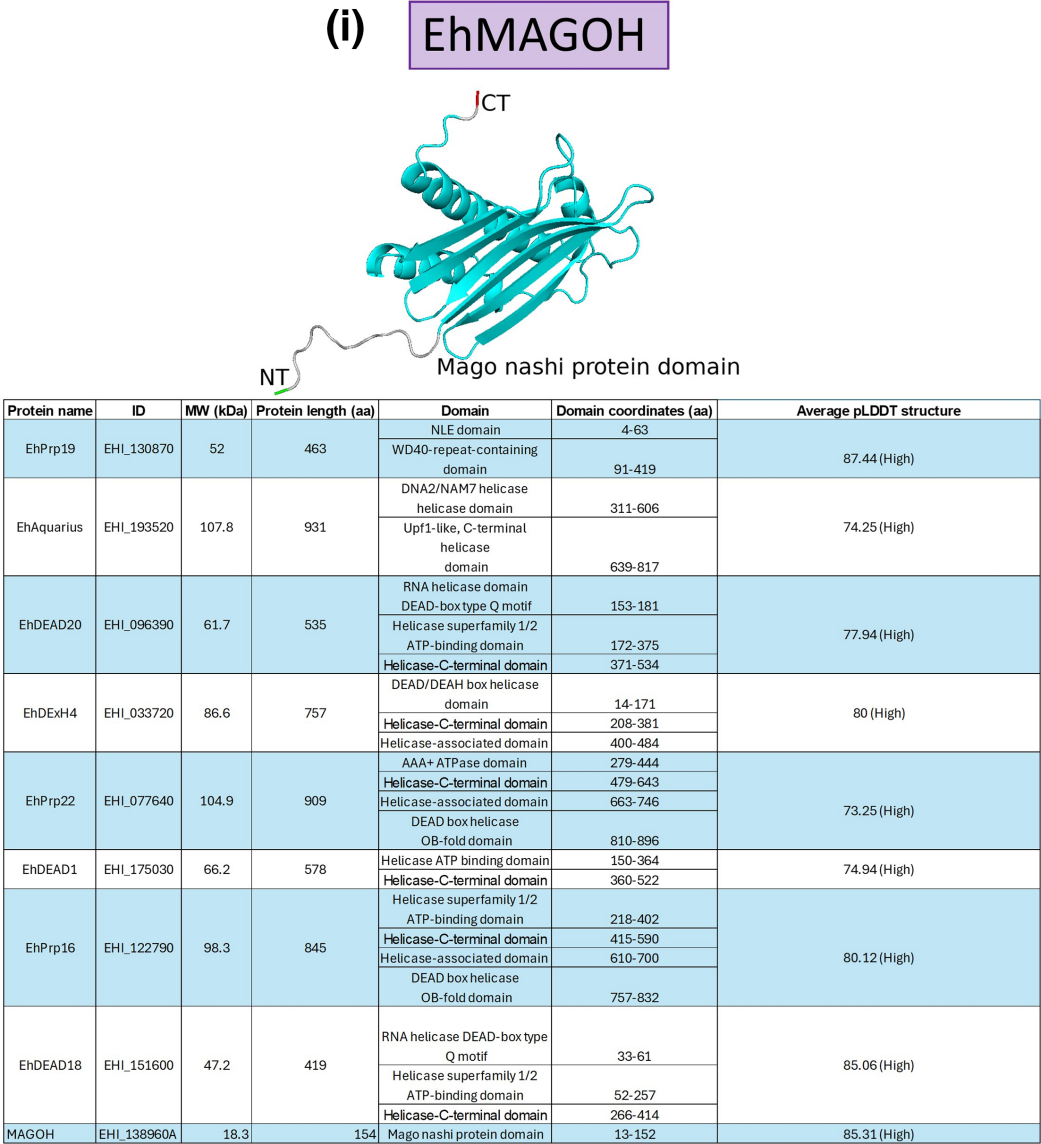
Proteins from the Complexes B/B^act^, Prp19/CDC5L, C and EJC of *E. histolytica,* showing their main domains.

Three components of the EhPrp19/CDC5L were found in *E. histolytica*: the WD domain-containing protein EhPrp19, essential for mature spliceosome stabilization and the first catalytic step of splicing, the RNA helicase EhAquarius orthologue required for spliceosomal repositioning, and the RNA helicase EhDEAD20 protein known as p68 (DDX5) (Fig. 7a–d). Additional evidence showed that EhSyf is also present in the EhPrp19/CDC5L [71]. All these factors are required before activation occurs. During B/B^act^ complex transition, we identified only one factor, the EhDExH4 Prp2 helicase from. EhDExH4 derived the conformational changes required for the B complex (Fig. 7e).

## Complex C

The catalytic step I spliceosome (complex C) is converted by Prp16 to the activated step II spliceosome (complex C*) that catalyses the ligation of the 5' exon to the 3' exon [77]. Currently, the structure of the *H. sapiens* and *S. cerevisiae* C* complex has been published using cryoEM [86,87]. The C* complex in humans catalyses step two, which generates the mRNA and the cut intron lariat; this reaction requires additional proteins, including SLU7, PRP18 and PRP22 [88,89]. Similar to *S. cerevisiae* spliceosomes, the lower core domain of C* contains U5 snRNP, U5 snRNA, PRP8, SNU114, U5-40K and U5 Sm core proteins [86]. In *S. cerevisiae*, the DEAH box ATPase Prp16 catalyses the conversion of complex C to the catalytically activated complex C* of step II, in which the second transesterification occurs [90].

In *E. histolytica*, C complex components include the Prp22 orthologue, EhDExH8, which disrupts RNA–RNA interactions, freeing the mRNA from U5; the EhDEAD1 helicase (Fig. 7f), which unwinds RNA hybrids; and the step II-factor Prp16 orthologue, EhDExH5, which facilitates exon–exon ligation (Fig. 7g). EhDExH8 and EhDExH5 perform quality controls of splicing. EhDExH8 rejects suboptimal 3'ss to ensure accurate gene expression, and EhDExH5 proofreads the 5'ss and the branch point, discarding substrates that are incorrectly branched.

A final component of the catalytic complex is the EJC. The EJC is a variable messenger RNP (mRNP) that is assembled to facilitate splicing. In metazoans, EJC facilitates essential functions in mRNA metabolism [91]. EJCs are composed of three invariant core factors and additional interacting proteins, and their assembly during intron splicing results in their binding to mRNA between 20 and 24 nucleotides upstream of exon–exon junctions due to their stable interaction with RNA [92]. The components of EJC by crystal structure during splicing are EIF4A3, RBM8A (Y14) and MAGOH. In mammals, we can find two homologous proteins, MAGOH and MAGOB. The DEAD-box protein EIF4A3 is the main RNA-binding component, while RBM8A and MAGOH form a heterodimer [93,94]. In *E. histolytica*, two components of the EJC, EhDEAD18 (Sub2p/UAP56; Fig. 7h) and EhMAGOH (Fig. 7i), were identified.

## Concluding remarks

Most splicing machinery factors have been predicted bioinformatically but not corroborated *in vivo*. Here, we discussed the main roles of the splicing factors previously reported in *E. histolytica* and compared their relevant domains and structures with those of their human or yeast counterparts. Several common features must be noted.

Most splicing factors are structurally conserved not only with humans and yeast but also throughout the Amoebozoa supergroup (Table S2), particularly in spliceosome core components like the U snRNAs, the EhSm and EhLSm proteins, EhPrp5, EhPrp8 and EhPrp19. This suggests a strong selective force towards full functional conservation as well. It must be stressed that very few orthologues are absent in certain amoeba species, although redundant functions of other splicing factors, easing the required roles, cannot be ruled out. This notion is strengthened by the fact that all EhDEAD/DExH helicases involved in spliceosome rearrangement, proofreading and pre-mRNA maturation are present in the Entamoeba spliceosome, and some of them are represented by multiple forms from different genes (the Prp43 orthologues EhDExH9, EhDExH13 and EhDExH7; the Brr2 orthologues EhDExH10/U5 and EhDExH1; and two EhCPR6 orthologues of 20 and 18 kDa, respectively).

Here, we showed that the structural conservation of splicing-related factors is also observed in free-living amoebas; therefore, it is likely that the splicing machinery arose early in the evolution of Amoebozoa, prior to or with the common ancestor that emerged in the aquatic environment. However, since the amino acid sequence conservation of human and *E. histolytica* Prp8 and Snu13 is almost absolute, horizontal transfer of some splicing factors must also be considered [95].

Although the lack of U1 snRNA has been observed in other organisms, efficient 5'ss activation occurs in Entamoeba introns; therefore, the U1 small nuclear proteins replace the functions of the U1 snRNA. Our unpublished observations suggest that U1C is the protein that binds to the 5'ss. However, the larger molecular mass of EhU1-70k (72 amino acids longer) relative to the human orthologue also suggests its involvement in this function, in addition to its interaction with Pol II CTD.

It appears that, in *E. histolytica*, initial splicing complex formation and activation require larger specific splicing factors, since EhSmF, EhU2A', EhU2AF2 and Eh65K/SAD1 (358, 606, 226 and 221 amino acids longer, respectively) are larger than their human counterparts. From these factors, only the impact of the extension of EhU2AF2 on splicing has been characterized. Previously, we have shown that the KH-QUA2 domain of EhU2AF2 interacts with the KH-QUA2 domain of EhSF1, stabilizing other (ULM-UHM) protein interactions between them and partially inhibiting the substitution of EhSF3B1-EhU2AF2 binding by the EhSF1-EhU2AF2 grip. This enhanced interaction blocks the transition from splicing complex E to A, leading to intron retention and partial splicing of the target introns and downregulating the parasite’s virulence. This particular function of EhU2AF2 constitutes the single example of a splicing factor-driven intron retention [70].

## Supplementary material

10.1099/mic.0.001722 Table S1.

## Abbreviations

BS: branch site
EJC: exon junction complex
mRNP: messenger RNP
pLDDT: predicted Local Distance Difference Test
pre-mRNA: precursor mRNA
PRMT5: protein arginine N-methyltransferase 5
Py: polypyrimidine
RRM: RNA-binding motif
SF1: splicing factor 1
SMN: survival motor neuron
SNAPc: snRNA-activating protein complex
snRNA: small nuclear RNA
snRNP: small nuclear ribonucleoprotein
SS: splice site
U2AF: U2 auxiliary factor
